# Usefulness of coil-assisted retrograde transvenous obliteration II (CARTO-II) for the treatment of ascending colonic varix: a case report

**DOI:** 10.1186/s42155-020-00187-2

**Published:** 2020-12-04

**Authors:** Hiroyuki Maeda, Ken Kageyama, Akira Yamamoto, Atsushi Jogo, Etsuji Sohgawa, Kazuki Matsushita, Kazuo Asano, Hiroki Yonezawa, Takehito Nota, Kazuki Murai, Satoyuki Ogawa, Yukio Miki

**Affiliations:** Department of Diagnostic and Interventional Radiology, Graduate School of Medicine, Osaka City University, 1-4-3 Asahimachi, Abenoku, Osaka, 545-8585 Japan

**Keywords:** Ectopic varices, Ascending colonic varices, B-RTO, CARTO-II

## Abstract

**Background:**

Colonic varices are rare among ectopic varices. A previous report demonstrated that once bleeding from colonic varices occurs, it can be fatal. Several treatments for colonic varices exist, including surgical, endoscopic, and endovascular treatments; however, management of colonic varices has not been standardized. For colonic varices, minimally invasive therapies would be desirable. Balloon-occluded retrograde transvenous obliteration (B-RTO) is one of the treatment options for colonic varices to prevent their rupture. Two cases of successful conventional B-RTO for these varices have already been reported. However, B-RTO using coil-assisted retrograde transvenous obliteration II (CARTO-II) procedure for these varices has not been reported.

**Case presentation:**

A 71-year-old male patient had liver cirrhosis caused by hepatitis C virus infection. A varix was located at the ascending colon, which was coincidentally found on colonic endoscopy. Contrast-enhanced computed tomography (CT) showed that the feeder vein was the ileocolic vein and that the main draining vein was the right renal vein. Physicians concluded that treatment was required to avoid the risk of death from massive bleeding due to varix rupture. However, endoscopic and surgical treatments were difficult due to the anatomical location of the varix and the high risk of operative compilations, respectively. This ascending colonic varix was treated by balloon-occluded retrograde transvenous obliteration (B-RTO) using coil-assisted retrograde transvenous obliteration II (CARTO-II) procedure via the right renal vein. There were no complications during the procedure and no recurrences for 36 months during long-term follow-up.

**Conclusions:**

CARTO-II can be one of the effective treatment techniques for ascending colonic varices.

## Background

Ectopic varices are defined as large portal-systemic venous collaterals occurring anywhere in the intestinal tract other than the esophageal region (Norton et al. 1998). Colonic varices are rare, accounting for 3.5% of all ectopic varices in a Japanese nationwide survey (Watanabe et al. 2010).

Variceal bleeding is a major concern with gastrointestinal varices. Ectopic varices are also an unusual cause of gastrointestinal hemorrhage and account for up to 5% of all variceal bleeding (Kinkhabwala et al. 1977). Bleeding from colonic varices is rare; the rate of their bleeding with liver cirrhosis is less than 9% (Hosking et al. 1989). Meanwhile, 14% of ectopic variceal bleeding occurs in the colon (Norton et al. 1998). When ectopic variceal bleeding occurs, the mortality rate reaches as high as 32.1%, including death in 25.0% of cases with colonic varices (Watanabe et al. 2010).

Management of colonic varices has not been standardized (Ko et al. 2013). There have been some reports of massive lower gastrointestinal bleeding caused by colonic varices treated by surgery (Lopes et al. 2006; Naef et al. 1998). However, most patients with colonic variceal bleeding have cirrhotic livers. In patients with liver cirrhosis, surgery leads to increased in-hospital mortality and prolonged hospital stays, even without portal hypertension (Csikesz et al. 2009). Thus, minimally invasive therapies have been sought for colonic varices (Ko et al. 2013). Balloon-occluded retrograde transvenous obliteration (B-RTO) is one of the treatment options for colonic varices and seems to be worth performing to prevent their rupture. Two cases of successful conventional B-RTO for ascending colonic varices have already been reported in the last 3 years (Matsumoto et al. 2018; Liu et al. 2020). However, to our knowledge, B-RTO using coil-assisted retrograde transvenous obliteration II (CARTO-II) procedure for colonic varices has not been reported.

## Case presentation

### Initial presentation

**Fig. 1:**
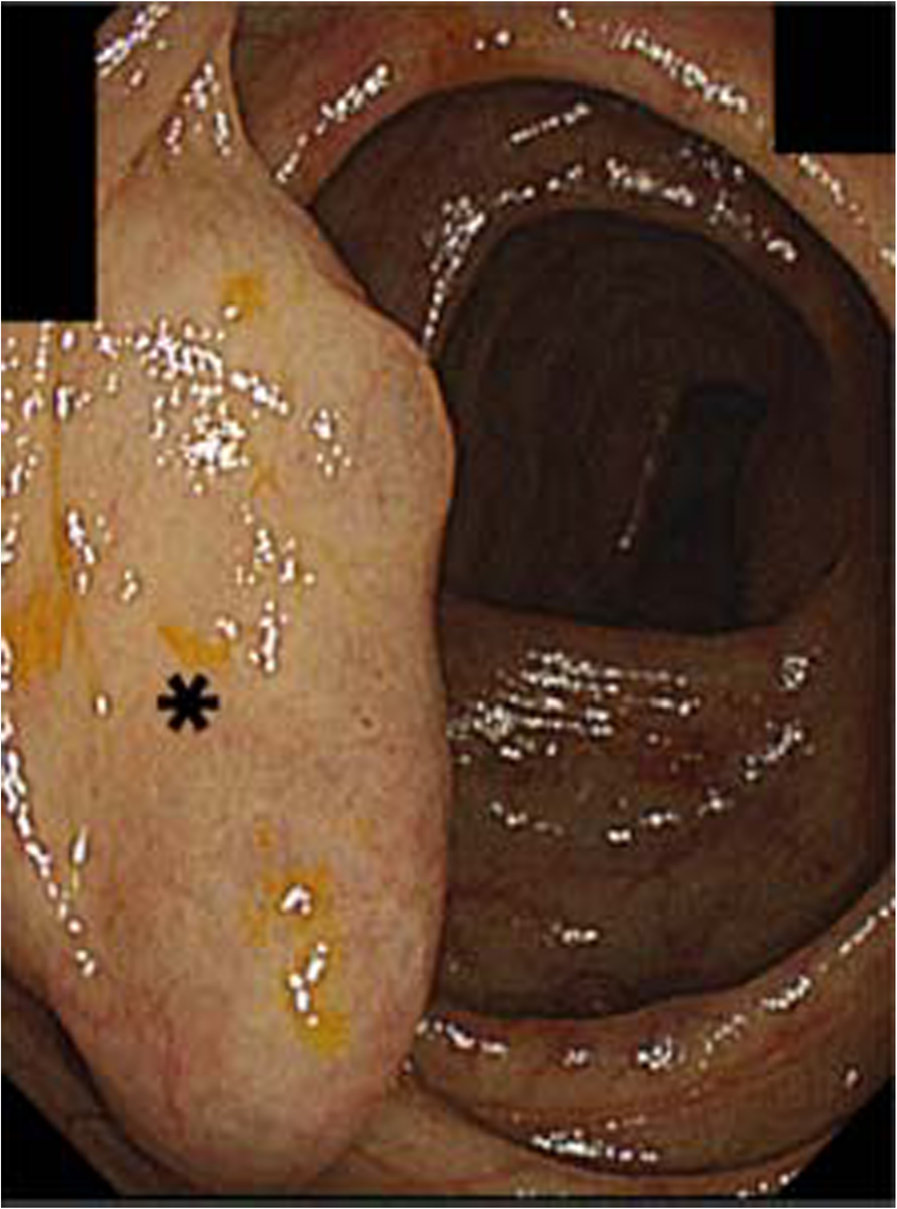
Endoscopic examination performed before B-RTO using CARTO-II. The endoscopy shows a varix (asterisk) located at the ascending colon

**Fig. 2:**
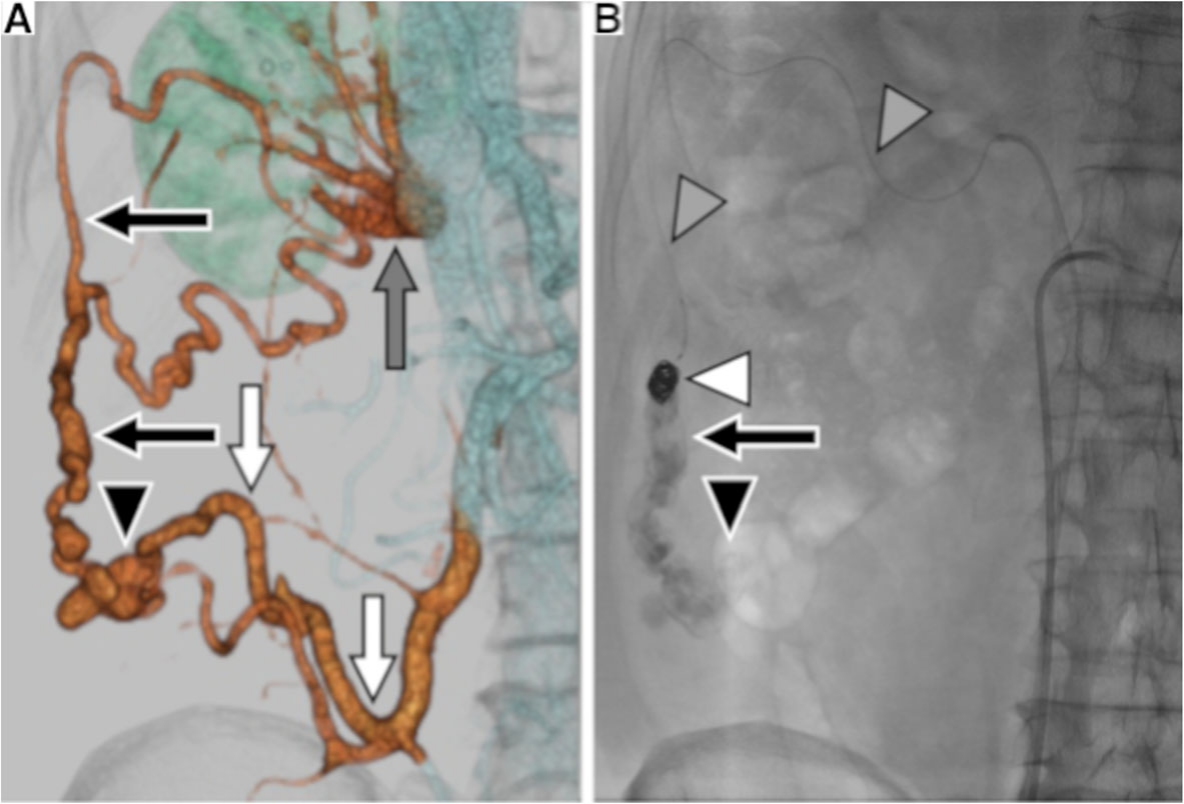
Overviews of anatomy and treatment. **a** Three-dimensional volume rendering image (VR image) reconstructed from contrast medium-enhanced CT. The image shows the ascending colonic varix (black arrowhead), the main draining vein (black arrows), and the ileocolic vein (white arrows) as a feeder vein. The gray arrow shows the right renal vein. **b** Overview X-ray image. After injection of the sclerosing agent, microcoils (white arrowhead) are inserted via the microballoon catheter (gray arrowheads). When the balloon is deflated, contrast medium is retained in the colonic varix (black arrowhead) and the draining vein (black arrow)

**Fig. 3:**
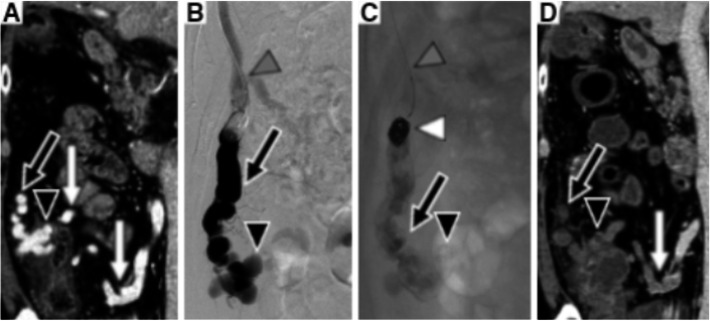
Close-up pictures of the ascending colonic varix. **a** Contrast medium-enhanced CT before B-RTO shows the ascending colonic varix (black arrowhead), the main draining vein (black arrow), and the ileocolic vein (white arrows) as a feeder vein. **b** B-RTV from the main draining vein (black arrow) shows the ascending colonic varix (black arrowhead) and microballoon catheter (gray arrowhead). **c** After injection of the sclerosing agent, microcoils (white arrowhead) are inserted via the microballoon catheter (gray arrowhead). When the balloon is deflated, contrast medium is retained in the colonic varix (black arrowhead) and the draining vein (black arrow). **c** corresponds to the enlarged image of Fig. 2b. **d** The day after B-RTO, contrast medium-enhanced CT shows no enhancement of the draining vein (black arrow), the colonic varix (black arrowhead), and the ileocolic vein (white arrow)

A 71-year-old male patient had liver cirrhosis caused by hepatitis C virus infection. A varix located at the ascending colon was coincidentally found on colonic endoscopy (Fig. 1). This case was finally diagnosed as a colonic varix on contrast-enhanced computed tomography (CT). CT images showed that the feeder vein was the ileocolic vein and that the main draining vein was the right renal vein (Figs. 2a and 3a). The patient did not have anemia due to melena or rectorrhagia. However, once bleeding from colonic varices occurs, the mortality rate is very high. Since endoscopic therapy is difficult to perform for a colonic varix from the anatomical perspective, and surgery has a high risk for patients with liver cirrhosis, endovascular treatment was considered reasonable.

### Interventional procedure

A 4.5-Fr 60-cm guiding sheath (Parent plus 45, Medikit, Tokyo, Japan) was inserted into the right renal vein via the right femoral vein under local anesthesia. Subsequently, a 3.3-Fr coaxial balloon catheter with a 10-mm balloon (Masamune, Fuji Systems, Tokyo, Japan) was inserted into the draining vein near the varix via the right renal vein through the guiding sheath. Then, balloon-occluded retrograde transvenous venography (B-RTV) was performed. When B-RTV showed contrast medium retention in the ascending colonic varix (Fig. 3b), the sclerosing agent consisted of 5% ethanolamine oleate iopamidol (EOI) mixed with 10% ethanolamine oleate (Oldamin, ASKA Pharmaceutical, Osaka, Japan), and the same volume of nonionic contrast medium (iopamidol 300 mg I/mL, Iopamiron 300; Bayer Schering Pharma, Osaka, Japan) was injected via the balloon catheter until the varix and the feeder vein were visualized by EOI. We applied CARTO-II procedure to this treatment (Yamamoto et al. 2020). After finishing injection of sclerosant, two microcoils (Tornado, Cook, Bloomington, IN, USA; Trufill, Cordis, Miami, FL, USA) were inserted into the drainage vein between the varix and the right renal vein through the microballoon catheter to prevent migration of sclerosant. When the balloon was deflated after coil embolization, we confirmed the sclerosing agent was still retained in the varix on X-ray (Figs. 2b and 3c). Just after the procedure, the microballoon catheter was removed. It took 107 min to complete the procedure without intra-operative complications.

### Follow-up

After CARTO-II, CT showed that obliteration was complete and successful without postoperative complications in this case (Fig. 3d). There was no exacerbation of portal hypertension, such as emergence of ascites and other varices, during the follow-up period. Ten months after the treatment, endoscopic follow-up examination showed that the varix had disappeared completely and returned to normal mucosa. The varix did not recur or bleed during the 36-month follow-up.

## Conclusions

This is the first report of B-RTO using CARTO-II for an ascending colonic varix. B-RTO has been performed for various ectopic varices, such as duodenal, stomal, and small intestinal varices, as well as colonic varices (Watanabe et al. 2010). There have been only two reports of B-RTO for an ascending colonic varix (Matsumoto et al. 2018; Liu et al. 2020). In these reports, conventional B-RTO was performed via the right renal vein. Similarly, in the present case, the varix was also located at the ascending colon, and B-RTO was performed via the same route. The present report reconfirmed the importance of B-RTO for ascending colonic varices by demonstrating no recurrence or complications during long-term follow-up. Furthermore, this report showed that CARTO-II procedure could be also applied for ascending colonic varices.

The physiological normal connections between the portal and the systemic veins have been known to portosystemic anastomoses. The recanalization of these embryonic channels results from portal hypertension, which causes an inflow from the high-pressure portal system to low-pressure venous systems (Sharma and Rameshbabu 2012). The venous blood from the right colon drains into the right gonadal, right renal, and right lumbar veins as draining veins (Sharma and Rameshbabu 2012). The feeding veins flowing into the right colon are the ileocolic, right colic, and middle colic veins. In the present case, the draining vein was the right renal vein and the feeder vein was the ileocolic vein.

Recently, modified versions of B-RTO have been developed, such as CARTO and plug-assisted retrograde transvenous obliteration (PARTO) (Lee et al. 2014; Gwon et al. 2013). CARTO procedure has two subtypes: CARTO or CARTO-I, and CARTO-II (Kim et al. 2018; Yamamoto et al. 2020). In CARTO, coil embolization of the draining vein is performed first, prior to injection of sclerosant, without balloon occlusion, whereas in CARTO-II, coil embolization of the draining vein is performed after injection of sclerosant under balloon occlusion. The balloon catheter is finally deflated and removed after the procedure in CARTO-II (Yamamoto et al. 2020). A recent study of CARTO-II demonstrated that this modified technique has two advantages compared to the original CARTO: keeping cost down and saving time (Yamamoto et al. 2020). One advantage derives from the use of a smaller number of coils than CARTO, the other from the lack of any need to wait for complete occlusion before injecting sclerosants. In the present case, only two microcoils were used to embolize the draining vein, and it took less than 2 h to complete the procedure using CARTO-II. With respect to the number of coils used and the procedure length, this case was similar to the previous study of CARTO-II (Yamamoto et al. 2020). Figure 2a shows that the draining vein was very tortuous and long to reach near the varix. PARTO would be difficult for treating the ascending colonic varix due to the difficulty of delivering a plug through the tortuous vein (Kim et al. 2018). CARTO-II procedure might be applied for other ectopic varices with tortuous veins, including ascending colonic varices. From an anatomical viewpoint, B-RTO using CARTO-II would be effective for ascending colonic varices.

BRTO using N-butyl-2-cyanoacrylate (NBCA) is one of the treatment options for varix therapy (Nakai et al. 2018). NBCA is a liquid sclerosing agent that polymerizes immediately on contact with anions in the blood (Nakai et al. 2018). Injection of a mixture of NBCA is more difficult than that of EOI, because NBCA causes adherence of the balloon catheter, microcatheter, and the blood vessel wall (Okahara et al. 2012). In this regard, the use of NBCA for BRTO requires considerable technical skill and experience.

An endoscope may not always reach the ascending colon due to its anatomical location (Hitoshi and Fukuji 1989). In the case of lower gastrointestinal bleeding, colonoscopy is ineffective because of poor visibility (Lopes et al. 2006). When the endoscope does not reach the ascending colonic varix, B-RTO using CARTO-II might be an alternative treatment.

In conclusion, this successful case suggests that B-RTO using CARTO-II can be one of the effective treatment techniques for ascending colonic varices.

## Data Availability

The relevant data have been included in the manuscript. The datasets used and analyzed during the current study are available from the corresponding author on reasonable request.

## Abbreviations

B-RTO: Balloon-occluded retrograde transvenous obliteration
CARTO: Coil-assisted retrograde transvenous obliteration
CARTO-II: Coil-assisted retrograde transvenous obliteration II
CT: Computed tomography
B-RTV: Balloon-occluded retrograde transvenous venography
EOI: Ethanolamine oleate iopamidol
PARTO: Plug-assisted retrograde transvenous obliteration
NBCA: N-butyl-2-cyanoacrylate
VR: Volume rendering
